# Integrating Text Structure Instruction in Science Education: A Design-Based Study

**DOI:** 10.1080/1046560X.2024.2373548

**Published:** 2024-08-07

**Authors:** Hilde S. Kooiker-den Boer, Ted J. M. Sanders, Jacqueline Evers-Vermeul

**Affiliations:** a Institute for Language Sciences, Utrecht University, Utrecht, The Netherlands; b Department of Health, Education & Wellbeing, HZ University of Applied Sciences, Vlissingen, The Netherlands

**Keywords:** Design-based research, reading comprehension, science-and-literacy programs, teacher professionalization, text structure instruction

## Abstract

Integrated science-and-literacy programs have proven to positively affect both language proficiency and science knowledge. Because making connections is important in both text comprehension and understanding the disciplinary core ideas taught in science, it seems worthwhile to explore the potential of integrating text structure instruction in science education. Therefore, we conducted a design-based research (DBR) in collaboration with teachers in the upper levels of primary education in the Netherlands. A set of four design principles directed both the design process and the analysis of this process. Research questions were aimed at the viability of these principles and at gaining knowledge about the application of DBR within the field of an integrated science curriculum. The study demonstrates the potential of DBR as a vehicle for translating research outcomes into educational practice. The four design principles eventually resulted in materials that worked well in grades 4–6. Still, since several design principles were new to the teachers, the design task was a challenge to the teachers and required support by the researchers. Especially the selection of suitable texts proved difficult. The study yields insights and recommendations for future DBR studies in the field of science-and-literacy integrated education. Given the abundance of cross-cutting concepts that can be tied to specific text structures, there is ample room for the development of integrated materials.

## Introduction

For decades, researchers, teachers and educational experts have shown an ongoing interest in the benefits of integrated instruction of literacy in the content areas. Multiple meta-analyses demonstrate that integrated science-and-literacy programs predominantly positively affect language proficiency (vocabulary, comprehension and writing skills) and science knowledge, and also improve attitudes toward reading and science (Bradbury, 2014; Graham et al., 2020; Hwang et al., 2022).

The combination of science teaching and language instruction seems recommendable. First, reading and writing skills are part of the work of scientists, and hence also need to be part of the science curriculum (Bradbury, 2014; Cervetti et al., 2012). The field of science has its own academic language: vocabulary, as well as specific discourse patterns not present in students’ everyday language, but needed for learning (Huerta & Garza, 2019). Second, working with authentic language activities is very important for promoting language proficiency (Purcell-Gates et al., 2007). Inquiry-based learning within science education offers many opportunities for such authentic language tasks, for example aimed at acquiring knowledge. Being engaged in knowledge building while reading a text increases interest and intrinsic motivation for reading, and thereby promotes deeper text processing (Jetton & Alexander, 2001; Wang & Guthrie, 2004). In addition, hands-on investigation offers many possibilities for authentic writing tasks, such as logs and reports (Cervetti et al., 2009).

At present, many science-and-literacy-integrated programs seem to focus mainly on vocabulary (Wright et al., 2016) and general reading and writing skills, which has yielded positive results (Bradbury, 2014; Graham et al., 2020; Hwang et al., 2022). However, it seems worth the effort to explore another option, related to the fact that both disciplines share cognitive processes throughout the research process. For example, metacognitive regulation, solving problems, and making connections are functions that, in reading as well as in inquiry learning, call for the construction of meaning (Bradbury, 2014; Cervetti et al., 2006). Hence, the focus of integrated programs could also be on the application of meaning making strategies such as drawing inferences and conclusions, making connections and recognizing relationships (Cervetti et al., 2006; Wallace & Coffey, 2019).

Teaching children about the most common text structures in expository texts, so that they can recognize the structure of a text and use this in processing and comprehending the text, has proven to be a powerful tool to foster reading proficiency (Bogaerds-Hazenberg et al., 2020; Hebert et al., 2016; Pyle et al., 2017). A coherent mental representation of a text, in fact, emerges by building relationships among the parts of a text, and between the text and one’s prior knowledge (van Dijk & Kintsch, 1983). This requires students to be able to recognize prototypical rhetorical structures consisting of specific coherence relations such as cause-effect or sequence at the sentence, paragraph and text level (Graesser et al., 2004; Ray & Meyer, 2011; Sanders et al., 1992; Sanders & Spooren, 2009).

The five most common text structures in expository texts—description, sequence, cause-and-effect, compare-and-contrast, and problem-and-solution (Meyer, 1975; see Table 1)—seem to match the disciplinary core ideas taught in science very well, especially crosscutting concepts such as *patterns* or *cause and effect* (Fick et al., 2022). In hands-on science activities, students can observe processes, or compare objects or phenomena, thereby experiencing the characteristics of these conceptual relations at first hand (Cervetti et al., 2006). For example, if students follow and compare the digestion process of a loaf of bread in various conditions, and discuss this process in terms of cause and effect, this can help build comprehension. Building upon this supposed synergistic relationship between science and literacy, we explored the potential of blending science education and text structure instruction.

**Table 1.** Description, signaling words, and example of five common text structures.

Text structure	Example
**Description:** Aspects of a topic *Signaling words*: For example, characteristics are	***Mosses*** *Mosses can be found everywhere: in the dunes, high in the mountains or just in your garden. Many mosses grow in forests, on rotting wood, or on the ground. They do not have real roots, but take the water directly from the air. There are nearly 10.000 varieties of moss! Moss is the oldest plant species on earth*. (*Alles-in-1*, grade 5/6, p. 63)
**Sequence:** Time-ordered collection of ideas or events *Signaling words*: First, second, before, dates	***Who eats what?*** *The caterpillar eats the leaves of a cabbage plant. A bird eats the caterpillar. The bird dies and is eaten by benthic animals and fungi. The minerals that remain are used by the plant to grow. The plant is again eaten by the caterpillar. And you can go on like this for a very long time. A cycle of food is created*. (*Naut*, grade 5, p. 32)
**Comparison:** Ideas related by differences and/or similarities *Signaling words*: The same as, instead, have in common	***Transparant*** *Ice, glass and plastic are a bit alike. You can see through all three materials. Yet they behave very differently. If you jump on a thin plate of ice, it will break. This also happens with glass, but not with plastic. Ice is more likely to melt than plastic*. (*Naut*, grade 4, p. 34)
**Cause-effect:** Main ideas organized into cause and effect parts *Signaling words*: Due to, because, consequence	***With strings attached to the sun*** *The sun is more than a million times the size of the earth and about 300.000 times as heavy. With all that gravity, the sun is pulling the planets. It seems as if they are on a string from the sun. The planets therefore revolve around the sun. This way the solar system stays neatly together*. (*Naut*, grade 4, p. 98)
**Problem-solution:** Main ideas organized in problem part and solution part *Signaling words*: Difficulty, ways to prevent, problem, solution	***Air in bottles*** *We are constantly breathing in and out. To be able to dive for an hour, you need a room full of air. Of course you can’t take that with you underwater. Fortunately, air is gas. You can compress gases. The amount of air in a room is made 100 times smaller. So it fits in the bottle on the back of the diver*. (*Naut*, grade 4, p. 36)

In primary education in the Netherlands, integrating science and literacy is hardly put into practice (Gresnigt, 2018), and text structure instruction also receives little attention (Bogaerds-Hazenberg et al., 2017, 2022; Kooiker den Boer et al., 2023). Furthermore, many teachers lack knowledge about text structure and text structure instruction (Beerwinkle et al., 2018; Bogaerds-Hazenberg et al., 2019; Reutzel et al., 2016), and the curriculum of teacher education in this area insufficiently aligns with the body of knowledge from reading research (Kooiker den Boer et al., 2019).

A research methodology that can both be helpful to close the research-practice gap and contribute to teacher professionalization is design-based research (DBR). In DBR educational interventions are designed, tested and evaluated systematically and based on scientific insights. The cyclical approach of DBR provides both practical and theoretical knowledge about the effective components of the design (Bakker, 2018; McKenney & Reeves, 2018).

Cross-disciplinary collaborations between teachers and researchers, such as within DBR, can be very effective (Ormel et al., 2012; Seidenberg et al., 2020; Vanderlinde & van Braak, 2010), since teachers can bring in knowledge of their students and affordances and constraints of their particular educational context (Fazio & Gallagher, 2018; McFadden & Roehrig, 2017). DBR can also generate teacher professionalization. Co-creating teaching materials provides teachers with opportunities to focus on research-based knowledge on student-learning of content, and to actively reflect on their practice (Binkhorst et al., 2015; Ingvarson et al., 2005).

Hence, we conducted a DBR aimed at the development and testing of teaching materials. Although this took place within the context of Dutch primary education, we expect the generated insights to be applicable to the development of science education in other countries as well, and to future DBR projects in the field of science-and-literacy integrated education.

A set of four design principles derived from scientific research directed both the design process and our analysis of this design process (see the next section). The following research questions guided our study:

RQ 1. How viable are the design principles related to integrated science-and-literacy education?

RQ 2. What do we learn from teachers’ experiences about the challenges and benefits of conducting design-based research in the field of integrated science-and-literacy education?

## Design principles

The purpose of our DBR was to develop science-and-literacy-integrated teaching materials with a focus on text structure instruction and, since the study took place in the context of Dutch primary education, aimed at language proficiency of Dutch. In terms of the integration staircase, which orders different types of integration in terms of complexity (Gresnigt et al., 2014), the approach in our study can be characterized as interdisciplinary integration. At this level of integration, two or more school subjects are organized around the same theme or topic, but the disciplines preserve their identity. Based on insights from previous research on reading comprehension and integrated programs, we formulated four design principles (DPs), which we will explain in the next paragraphs.

### Selecting concepts

*DP1. Select concepts that enable hands-on activities and evoke understanding processes and/or understanding causal relationships and/or comparing and classifying content.*

Instruction about text structures such as the ones in Table 1 seems compatible with the three-dimensional learning described in the Framework for K-12 Science Education (National Research Council, 2012). This entails understanding of *core conceptual ideas* occurs through engagement in *science activities* and by using *crosscutting concepts* as lenses or bridges that can support students’ science sensemaking (Fick et al., 2022; Rivet et al., 2016). The connection aimed at in DP1 is supposed to work two ways. First, during science activities students can observe sequential processes, compare objects or phenomena, or predict the effects of certain operations, and experience the characteristics of these coherence relations at first hand (Cervetti et al., 2006). Second, learning to recognize the structure of texts within the context of science education can support students’ conceptual understanding (Montelongo et al., 2010) and thereby facilitate the use of the crosscutting concepts (Fick et al., 2022).

### Hands-on activities

*DP2. Organize hands-on activities that facilitate the comprehension of the concept and its context.*

Experiencing science concepts through hands-on activities is motivating and promotes active thinking and discourse around activities. Several scientists agree that science and literacy share a set of meaning making strategies and are thus synergistically related (Cervetti et al., 2006; Guthrie et al., 1999; Stoddart et al., 2002). Working on these strategies in both inquiry and literacy activities can reinforce conceptual understanding as well as language proficiency (Bradbury, 2014; Huntley, 1998; Pratt & Pratt, 2004). Extending this premise to text structure instruction, it is important to not only familiarize students with coherence relations such as sequence, or cause-effect during reading lessons, but also let them perceive and explicate such relations in real-world experiences.

Inquiry activities offer many opportunities for authentic reading and writing activities (Bradbury, 2014; Stoddart et al., 2002). Reading can support students in understanding scientific concepts and phenomena observed in first-hand investigation, but texts can also guide the process of inquiry (Cervetti et al., 2006). In addition, hands-on activities provide many opportunities for purposeful writing tasks such as writing reports or logs (Huerta & Garza, 2019; Tilson et al., 2010). Creating such a motivational context will enhance reading engagement, thereby promoting deep text processing, which will benefit text comprehension (Guthrie et al., 1999; Toste et al., 2020).

### Authentic reading and writing activities aimed at text structure instruction

*DP3. Design authentic reading and writing activities targeting text structure instruction and use, when suitable, graphic organizers.*

Several meta-analyses demonstrate positive effects of text structure instruction on text comprehension of both narrative and expository texts, even in young children (Bogaerds-Hazenberg et al., 2020; Hebert et al., 2016; Pyle et al., 2017). Knowledge about text structure can enhance insight in how the author of a text presents and organizes information, and can thus free up memory and processing resources which allows the reader to focus on the content of the text. It also promotes deciding, which information is important. Markers of text structure such as signaling words can cue text structures and help readers build a coherent text representation (Meyer & Ray, 2011; Sanders et al., 2007; Sanders & Noordman, 2000; van Silfhout et al., 2015).

Many reading researchers argue for balanced comprehension instruction that includes both explicit instruction and opportunities for practicing reading and writing in authentic literacy activities (Duke & Pearson, 2009; Rooijackers, 2023). Effective text structure instruction initially calls for explicit instruction; teachers should model the use of texts structures in reading and make use of scaffolding to facilitate students independent use of the text structure during the reading process (Hebert et al., 2016; Meyer & Ray, 2011; Purcell-Gates et al., 2007; Pyle et al., 2017). By providing reading and writing activities aimed at text structure knowledge within the content area students can experience the utility of text structure knowledge in authentic language activities (Wijekumar et al., 2017).

A specific tool frequently applied in text structure instruction is the use of graphic organizers that visually represent the text organization, which is particularly effective when students actively fill out these organizers, for instance for text summarization or in prewriting activities (Bogaerds-Hazenberg et al., 2020; Pyle et al., 2017). Within the context of science, graphic organizers can help consolidate conceptual knowledge of the subject (O’Donnell et al., 2002; Romance & Vitale, 1999).

### Declarative, procedural and conditional knowledge and model texts

*DP4. Teach declarative, procedural and conditional knowledge about text structure in reading and writing activities and make use of model texts with a clear structure.*

Good readers are active readers who make use of metacognitive knowledge about the reading process (Duke & Pearson, 2009; Iwai, 2011). Helping students acquire the strategies and processes used by good readers can enhance text comprehension of less-skilled readers (Duke & Pearson, 2009). Such strategy instruction should pay attention to three types of knowledge: declarative, procedural and conditional knowledge (Lorch et al., 1993; Paris et al., 1983; Rahmat et al., 2022). Declarative knowledge in the context of reading means factual knowledge (the what). In text structure instruction this may include knowledge about the features of various text structures, or about signaling words that highlight specific coherence relations. Procedural knowledge is knowledge about how certain reading activities or strategies have to be executed (the how), for example how to make use of a graphic organizer to visualize the structure of an expository text. Thus, declarative and procedural knowledge pertain primarily to the knowledge required to perform the reading task. Conditional knowledge concerns knowing when and for what purpose it is best to use a particular reading strategy (the when and why). For example, students may be able to summarize a text using a graphic organizer, but also need to know in what type of activities this will be a helpful strategy and what they can gain from applying it. This type of knowledge supports students to purposefully apply knowledge about reading strategies in various situations that call for text processing (Bogaerds-Hazenberg et al., 2020; Iwai, 2011; Rahmat et al., 2022).

When introducing a new text structure, teachers will have to draw students’ attention to the specific features of the structure (declarative knowledge) and model how they use knowledge about this text structure during reading or, for example, summarizing the text (procedural knowledge) (Meyer & Ray, 2011; Pyle et al., 2017). To this end, teachers need exemplary model texts that match the reading level of the students, cover a single top-level structure (Jones et al., 2016; Meyer & Ray, 2011), and incorporate signaling words that support identification of the text structure (van Silfhout et al., 2015). As students become more knowledgeable about various text structures, it is important that they also learn to apply this knowledge while reading authentic texts that often combine multiple text structures (conditional knowledge) (Duke et al., 2011; Jones et al., 2016). Therefore, in the teaching materials, we tried to balance declarative, procedural and conditional knowledge.

## Method

### Research context and participants

Being the initiators of the project, the researchers made use of their professional network to find schools willing to participate. Hence, we applied convenience sampling to form a design team. Initially two primary schools joined in. The participating teachers were interested in the research project as it aligned with educational developments at their schools. A third school became involved because a pre-service teacher joined in as part of her graduation assignment.

Throughout the project eight teachers and one pre-service teacher voluntarily participated in the design team. Four teachers taught in grade 4, two in grade 5 and two in grade 6. The pre-service teacher worked in a combined group of grades 3 and 4. During the first design cycle five participants performed the task of developing and testing the lessons, while the others tested the materials and provided feedback. Due to the COVID-19 pandemic we did not manage to complete the project in one school year (as was originally planned), but we were able to restart at the beginning of the next school year. This led to some changes in the design team, as three teachers dropped out and two others took their places. The total group of participating teachers had an average of 11.8 years of teaching experience (SD = 9.9).

Table 2 provides an overview of the design process. The project started with separate meetings in both schools (Meeting 1a and 1b); then five joint meetings were held, with a fairly long period between Meeting 4 and 5 due to the COVID-19 pandemic. Apart from Meeting 5, which was held online, meetings took place at one of the schools (with two teachers participating online during Meeting 6). During the meetings, the researchers explained the design principles, the design process of the lessons was discussed and participants reflected on their experiences. As Meeting 5 marked the relaunch of the project, the design principles were revisited and explained to introduce them to the newly participating teachers.

**Table 2.** Overview of the design process and types of data collected.

	Program of the meeting	Data collection
**Meeting 1** *On site*	Getting to know each other Exploring the scope and goals of the project Brainstorm about concepts	Audio recording 1a + 1b
**Design phase**	Teachers consider their concept choice	
**Meeting 2** *On site*	Clarification of the design principles Selecting a concept Working on a raw draft of the lesson series	Audio recording 2 Survey 1
**Design phase**	Teachers involved in the design process make a first draft of their lesson series	Logbook 1
**Meeting 3** *On site*	Exchange experiences during the design process Reflection on the application of design principles in the lessons Working in pairs on fine-tuning the design	Audio recording 3
**Test phase 1**	All teachers try out their lesson series	Logbook 2
**Meeting 4** *On site*	Exchange experiences during the first testcycle Reflection on the application of design principles in the lessons Evaluating the lessons and formulating points for improvement	Audio recording 4
**Design phase**	Researchers revise the teaching materials developed so far	
**Meeting 5** *Online*	Restart of the project Researchers present the revised teaching materials and ask for feedback	Audio recording 5
**Test phase 2**	Teachers try out the lesson series and researchers conduct lesson observations	Logbook 3 Lesson observations
**Meeting 6** *Hybrid*	Focus group discussion with an evaluation of the project	Audio recording 6 Survey 2

The first author initiated the project, moderated the group meetings, observed lessons and was the primary supervisor of the teachers. The second author acted as secondary supervisor and attended most of the meetings. In between sessions the researchers were available for teacher feedback or questions by mail contact. The study was granted ethics approval by the Faculty Ethics Assessment Committee of the Humanities Faculty of Utrecht University (reference number: 20-264-01).

### Data collection

In DBR data-collection is aimed at gathering information about the viability (practicality, relevance, sustainability), legitimacy (based on contemporary insights, consistency, coherence), and efficacy (do they yield the desired results) of the educational materials designed. As is common in DBR, our study contributes to three types of outputs: design principles, curricular products, and professional development of the participants involved (McKenney et al., 2006).

We applied methodological triangulation (Lincoln & Guba, 1985) by collecting a variety of data (see Table 3): audio recordings of group meetings, notes from lesson observations, logbooks and surveys. The teachers who designed the lessons filled out a logbook during the first design phase, and all teachers kept up a logbook during the first and second test cycle of the teaching materials. These logbooks provided information about preparation time and duration of the lessons, and about teachers’ satisfaction with the lessons in general and with respect to the implementation of the DPs. Furthermore, the teachers were asked about points of improvement for the lessons. During the first and last meeting, participants were asked to fill out a survey that included a mix of Likert-scale questions and open-ended questions about their familiarity with the subjects discussed, what new insights they had gained, and which elements of the DPs they had implemented in their own classroom practice. During the second test cycle, the first author observed two lessons from each teacher, noting the duration of the stages of the lesson, student participation and involvement, and the extent to which the lessons (*n* = 12) were carried out as presented in the manual.

**Table 3.** Survey 1 – means (and standard deviations) of familiarity with the topics discussed and understandability of information provided during meeting 2 (*n* = 6).

Topics	Familiarity with the topic (min. = 1; max. = 7)	Understandability of provided information (min. = 1; max. = 7)
Design-based research	5.00 (0.63)	5.50 (1.38)
Integration of subjects	5.17 (0.75)	5.83 (0.41)
Authentic reading and writing tasks	4.67 (0.52)	5.67 (0.52)
Text structure	4.17 (0.98)	5.33 (0.82)
Graphic organizers	5.00 (1.10)	5.33 (0.52)
Declarative, procedural and conditional knowledge	1.83 (1.17)	4.83 (1.47)

### Method of analysis

The audio recordings of the meetings were transcribed using on-line transcription software. The software package ATLAS.ti was used to select and code relevant chunks of these transcripts. In order to answer RQ1, we applied deductive coding to the data set (Linneberg & Korsgaard, 2019), taking the four design principles as a starting point. During the first coding cycle we noticed that DP3 and DP4 were too wide ranging, which is why we broke them down into smaller parts. This resulted in a final list of seven codes: 1) Select concepts, 2) Hands-on activities, 3a) Authentic reading and writing activities, 3b) Reading and writing activities aimed at text structure instruction, 3c) Make use of graphic organizers, 4a) Declarative, procedural and conditional knowledge, 4b) Make use of model texts with a clear structure. To gain insight into the process of introducing and implementing the DPs, we made an overview of the results per code.

In order to answer RQ2, we used the conceptual framework for Teacher Design Teams (Binkhorst et al., 2015) as a tool for understanding factors at the input, process or outcome level that can promote or interfere the effectiveness of a design project and thus be helpful to optimize the effectiveness of future projects. Since RQ2 focused on teachers’ experiences during the project, our analysis focused on the three evaluation domains of the outcome level *participants professional development*: satisfaction with the experience, teachers’ learning and teachers’ change of practice (Binkhorst et al., 2015; Guskey, 2002). We limited this analysis to teachers’ self-reported experiences during Meeting 6 and in Survey 1.

## Results

In this section, we first provide a general impression and an overview of the curricular products developed in the project. Then, in answer to RQ1 we report on the viability of the DPs, and describe what we learned about the challenges and benefits of conducting DBR (cf. RQ2).

### General impression and output of the project

During Meetings 1a, 1b and 2 the DPs were introduced and discussed. Table 3 shows how familiar teachers were with the topics and how understandable they found the information presented. The results of Survey 1 show that teachers were reasonably familiar with most of the subjects discussed during Meeting 1, with two exceptions: they were not very acquainted with the information provided about text structure, and the concepts *declarative*, *procedural* and *conditional knowledge* were completely new to them.

All teachers enthusiastically engaged in designing their lesson series. However, it appeared to be a challenge to apply all four DPs simultaneously. Table 4 shows the degree of difficulty teachers experienced in implementing each DP, and how satisfied they were with the quality of its implementation. The teachers could implement DP1 and DP3 without much difficulty. DP2 took more effort, but particularly DP4 was considered hard to apply.

**Table 4.** Logbook 1 – means (and standard deviations) of difficulty to take the DPs into account and satisfaction with their implementation in Test cycle 1.

DP	Difficulty in taking into account the DP (min. = 1; max. = 7)	Satisfaction with implementation of the DP (min. = 1; max. = 7)
DP1 (*n* = 5)	3.20 (1.30)	4.00 (0.71)
DP2 (*n* = 3)	4.67 (1.53)	4.33 (1.16)
DP3 (*n* = 4)	3.25 (1.89)	4.00 (1.41)
DP4 (*n* = 3)	6.00 (0.00)	4.33 (0.58)

In the continuation of the project we noticed that designing and testing the lessons took the teachers more time than estimated beforehand. At the time of Meeting 3 only one teacher had tested all the lessons she had developed. The others had only given one or two lessons. Unfortunately, the project was shut down shortly thereafter because of the COVID-19 pandemic. The researchers then decided to take over the design task and took charge of adapting the lesson series for a second cycle of testing, to ensure the progress of the project. There was much work to be done on making the teaching materials suitable for exchange between schools. For example, the teacher manuals were very brief and there was also still little consistency between lessons. Since teachers found it difficult to provide explicit instruction on text structures, supportive materials, such as model text and scripts for modeling, were added.

During Meeting 4, the modified lesson series were presented to the teachers for feedback. Then, all four lesson series were tested and one of the researchers conducted lesson observations. Table 5 summarizes the main findings of the observations during Test cycle 2. It shows that teachers did not always carry out text structure instruction as intended in the manual, which is why the learning goal was not always achieved either (see the previous section). Still, in general, students were able to perform the reading and writing activities. Most lessons turned out to be overloaded, teachers spent longer than scheduled or chose to skip activities. Table 6 presents the outcomes of the evaluative questions that teachers answered in their logbooks for each lesson.

**Table 5.** Lesson observations during Test cycle 2 – key features of the lessons, and number of lessons in which they were applied (*n* = 12).

Instructional features and student activities	yes	n.a.
**Instruction about text structure**		
Teacher pays attention to declarative knowledge about text structure	10	2
Teacher pays attention to procedural knowledge about text structure	6	2
Teacher pays attention to conditional knowledge about text structure	3	2
Teacher models reading the sample text	4	4
Teacher models use of the graphic organizer	5	4
**Reading activities**		
Students can work independently on the reading activity	9	3
Students can work with the graphic organizer	7	5
Students are engaged during the reading activity	9	3
**Writing activities**		
Students can work independently on the writing activity	5	7
Students can work with the graphic organizer	2	10
Students are engaged during the reading activity	5	7
**Learning goals and time schedule**		
Subject content goals were adequately addressed	11	0
Goals aimed at text structure knowledge are adequately addressed	9	0
Activities can be carried out in the time scheduled	3	0

**Table 6.** Logbook 3 – means (and standard deviations) of teacher evaluation of the lessons, teaching materials and own performance during Test cycle 2.

Instructional feature that was evaluated	Mean (SD) (min. = 1; max. = 7)
**Overall evaluation of the lessons**	
Overall working atmosphere (*n* = 35)	5.97 (0.86)
General satisfaction with the lesson (*n* = 35)	5.80 (0.80)
Level of the lesson for the students (*n* = 35)	4.43 (1.36)
Student engagement (*n* = 35)	5.97 (0.71)
**Satisfaction with the teaching materials**	
Hands-on activities (*n* = 23)	5.83 (1.37)
Reading activities (*n* = 24)	5.67 (0.82)
Writing activities (*n* = 27)	5.37 (1.18)
Use of graphic organizers (*n* = 27)	6.04 (0.76)
Texts (*n* = 23)	5.65 (1.03)
Teacher manual (*n* = 34)	6.00 (0.92)
**Satisfaction with own instructional performance**	
Working toward science content goals (*n* = 33)	5.88 (0.86)
Working toward reading and/or writing goals (*n* = 29)	5.90 (0.82)
Providing instruction about text structure (*n* = 28)	5.79 (0.83)

Table 7 provides an overview of the four lesson series after the second design cycle. Overall, teachers were satisfied with the teaching materials. The lessons were not too difficult but not too easy either. Teachers found the learning activities useful and were also satisfied with their own performance during the lessons. In Meeting 6 the quality of the teaching materials was discussed. Teachers especially appreciated the integration of reading, writing and hands-on activities in the lesson series, and were also very satisfied with the consistency between lessons. Furthermore, they indicated that they had been inexperienced with providing text structure instruction and were pleased with the scripts for modeling the reading of sample texts that were provided in the manuals. This general impression of the process and outcomes of the project indicates that the design principles turned out to be viable in classroom practice.

**Table 7.** Overview of the four lesson series after Test cycle 2.

Lesson series	Topic	Grades	Concepts	Text structures	Text titles	Hands-on activities
1	Electricity	3 and 4	Power circuits Conduction and isolation Sources of electricity Resources management Sustainability	Sequence Comparison Cause-effect Problem-solution	How is electricity made? To the fuel pump or charging station? (about electric cars) Coal-fired power plants: bad for the environment We take action!	Building power circuits Experiment with conduction and isolation
2	Sugar	3 and 4	Production processes Food relations Health	Sequences Comparison Cause-effect	From sugar beet to sugar bowl Beet or cane sugar? Sugar and your body	Examining several types of sugar Cooking applesauces with various sweeteners
3	Trash	5 and 6	Material characteristics Sustainability Nature conservation	Cause-effect Problem-solution	Why one material digests and another does not Plastic, a disaster for nature Away with the plastic soup!	Trash hunt Experiment with the degradation of a sandwich
4	Digestion and respiration	5 and 6	Metabolism Health Food relations	Sequence Comparison Cause-effect	From mouth to poop Your breathing, this is how it works One pellet is not the other Abdominal and chest breathing What smoking does to your lungs	Plucking out and analyzing owl pellets Modeling the respiration process with a plastic bottle

### DP1. Selecting concepts

The Dutch science curriculum for primary education offers a list of concepts that are supposed to be taught (van Graft & Klein Tank, 2018; Klein Tank, 2009). We invited the teachers to choose a topic from this list for their own lesson series. It took teachers little effort to apply DP1; they came up with many ideas, and Logbook 1 (Table 4) shows that they did not find this the most difficult design principle.

Transcripts of Meetings 1a and 1b show that teachers’ personal preferences and interests played an important role in the selection process:

I am also considering the topic *outer space*, but that’s mostly because I really like that myself. (Teacher 9, Meeting 1a)

They also made their own prior knowledge a consideration. Concepts about which they did not feel knowledgeable enough were rejected as topics for their lesson series. Further, we had to narrow the chosen topics because the original broad topics complicated the construction of a coherent set of learning goals that could be covered in six lessons. One of the teachers, for instance, started out with the topic *food*, but narrowed this to *sugar*.

It turned out that the science curriculum includes many concepts that offer opportunities for hands-on activities and allow for causal thinking, understanding processes or comparing content. The teachers were well capable of selecting appropriate concepts. From this we can conclude that DP1 is viable.

### DP2. Hands-on activities

During Meetings 1a, 1b and 2 teachers came up with many ideas for hands-on activities that tied in with the selected topics and concepts. However, organizing and preparing hands-on activities proved to be challenging; two teachers invited guest teachers for this purpose, and another teacher reported she had spent a lot of time testing an experiment only to discover it would not be feasible in their classroom. After they had conducted the first lessons with hands-on activities, all teachers were very enthusiastic about the high level of engagement these generated among their students:

During the first lesson we went on a trash hunt, exploring what trash they found in the neighborhood (…). Children were actually very surprised about the enormous amount of trash they found, but also about what it contained. That was a good lesson to follow up with a writing activity aimed at the problem-solution text structure. What can we do about it? The trash hunt had made them very enthusiastic. (Teacher 8, Meeting 6)

For Test cycle 2, researchers provided teachers with all materials for the hands-on activities. For reasons of transferability, the guest lessons were transformed into lessons teachers could deliver independently. The teachers were comfortable with this and contributed practical suggestions in the logbooks to further improve these activities. Survey 2 (Table 8) shows that, of all elements in the lesson series, teachers felt most confident about guiding hands-on activities. When asked at the end of the project what had been the three most important eye-openers for them in designing and testing the teaching materials, two teachers mentioned the engagement-enhancing influence of the hands-on activities, and one called the combination of inquiry and writing activities. During the design process, we noticed that there were many opportunities to connect authentic language activities to hands-on learning (Table 7), and that students were highly motivated during inquiry learning but also during language activities (Table 5).

**Table 8.** Survey 2 – means (and standard deviations) of teachers’ self-efficacy regarding the elements of the lesson series (*n* = 6).

Instructional features	Mean (SD) (min. = 1; max = 7)
Linking language goals and subject content goals	4.83 (0.98)
Devising authentic reading and writing activities	4.67 (0.52)
Providing instruction about text structure	5.50 (0.55)
Drawing attention to declarative knowledge about text structure	5.17 (0.41)
Drawing attention to procedural knowledge about text structure	5.17 (0.41)
Drawing attention to conditional knowledge about text structure	4.83 (0.75)
Using graphic organizers	5.67 (0.52)
Guiding hands-on activities	6.17 (0.75)

It is difficult to determine on the basis of the collected data to what extent teachers deliberately and explicitly paid attention to related crosscutting concepts during hands-on activities. Yet we think there was a clear link to the concepts in most of the hands-on activities. For example, building an electrical circuit and experimenting with various materials helped students to understand the concepts of conduction and insulation. Preparing as well as carrying out hands-on activities also turned out to be neither complicated nor very time-consuming. Short and simple activities such as discovering various types of sugar, or observing the degradation process of a sandwich, are very motivating and can evoke questions related to underlying concepts such as production processes or material characteristics. As for DP2, we can conclude that it proved viable. Teachers felt fairly confident about this DP.

### DP3. Authentic reading and writing activities, text structure and graphic organizers

During Meeting 2 the five common text structures were introduced (Table 1). The teachers indicated that the step-by-step teaching of knowledge about these text structures was not part of the teaching programs they normally used, and thus were new to both them and the students. They were familiar with the use of authentic reading tasks, but this was not something they applied on a daily basis. Some of the teachers already worked with graphic organizers in their lessons regularly. Table 3 provides further information about how familiar teachers felt to these topics, and how understandable the information provided during Meeting 2 was to them.

The first draft of the teaching materials developed by the teachers shows that their initial focus was primarily on introducing text structures and using graphic organizers for summarization. In doing so, they most often chose texts with a sequential or a comparison structure. Table 4 shows that they did not find DP3 the most difficult design principle to apply. Yet, in the first draft of their lesson series they often had not considered the authenticity of the reading and writing activities. Suggestions provided by the researchers and sharing of ideas during the meetings were helpful to enhance authenticity. In one of the reading lessons, for example, students had to read a comparison text about electric cars and cars running on gasoline, and summarize the text in a Venn diagram. We suggested an activity where students had to use the information from the text to create a role-play featuring a car salesman and a customer. In the modified lesson series for Test cycle 2, the researchers added elements to enhance authenticity even more, for instance by making writing activities more audience-oriented or by having students process the content of a text in a creative way.

During the meetings the teachers reported that the authenticity of the assignments contributed greatly to student engagement. For example, writing a manual for building a circuit generated great involvement:

They wrote a manual for building a circuit, and children were very critical towards one another. They said: you forgot the fitting, so now I can’t do it at all, because nowhere does it say that I need a fitting. So, it was a real pleasure to watch them looking at each other’s instructions very critically. (Teacher 1. Meeting 6)

Teachers experienced that working with graphic organizers helped students process the text deeply, although they noticed that students struggled with the use of graphic organizers for text summarization:

I expected them to figure out those organizers faster (…). These seem very easy to us, but for children it can still be a bridge too far, so you have to show them how to do it step by step. (Teacher 8. Meeting 6)

During the lesson observations, we noticed that teachers often did not explicitly model how to use a graphic organizer for text summarization. As a consequence, students often had questions or needed some support after instruction (Table 5).

DP3 certainly seems viable in educational practice. The content learning goals could easily be connected to various text structures, and text structures in turn offered starting points for authentic language activities such as making a presentation about the problems and solutions concerning trash, or writing a manual for building a power circuit. However, teachers struggled with providing text structure instruction. We will further elaborate on this in the next section. Although teachers stated that the use of graphic organizers requires more extensive instruction and guided practice than was currently offered, their added value was clear. Graphic organizers turned out to be helpful when summarizing a text as well as in preparing a writing assignment.

### DP4a. Declarative, procedural and conditional knowledge

The concept of teaching declarative, procedural and conditional knowledge appeared to be completely new to all teachers. After clarification, teachers were able to relate the concepts to their own teaching practice and the teaching materials they normally use. The results of Survey 1 (Table 3) also confirm teachers’ lack of knowledge of this topic.

Logbook 1 (Table 4) and the first draft of the lesson series show that teachers struggled to apply this newly acquired knowledge in their lessons. The learning goals they included in the manuals were rather broad, mainly focused on declarative and procedural knowledge, and hardly on conditional knowledge. In the lesson materials, most attention was being paid to declarative knowledge about a specific text structure: its main features and typical keywords.

After Test cycle 1, the researchers made several adjustments to the teaching materials to support teachers in addressing all three knowledge types more intentionally. We reformulated the lesson goals and indicated for each goal whether it was aimed at declarative, procedural or conditional knowledge. To deepen declarative and procedural knowledge about text structure, we made sample texts and scripts for modeling that teachers could use during reading instruction. Additionally, we provided posters with a short description of a specific structure, keywords and a matching graphic organizer. To facilitate working on conditional knowledge, we added short activities to the lessons that challenged students to apply the newly acquired knowledge about text structure. For example: *You now know the features of a text with a sequential structure. Can you think of other topics for which you might expect a text with a sequential structure?* In addition, we added writing activities that required students to determine the most suitable text structure.

During the lesson observations in Test cycle 2, we checked to what extent teachers paid attention to declarative, procedural and conditional knowledge (Table 5). Not surprisingly, teachers paid most attention to declarative knowledge. Procedural knowledge received less attention. For example, teachers did not always model how to make use of the text structure while reading a text, or how to fill out a graphic organizer. We observed very few sound examples of targeted instruction on conditional knowledge. Since many lessons took more time than scheduled, not all planned activities could always be done. In some cases, it were precisely the activities aimed at conditional knowledge that teachers left out.

During Meeting 6, the DPs were evaluated and teachers mentioned that conditional knowledge indeed had not received sufficient attention:

I think they haven’t really mastered conditional knowledge yet. In this series it only came up once and then I think, to what extent can they do that if you offer it once? (…) When they had to write a text I did ask about it: Why did you choose this text structure? They could indicate that, but I wonder whether it was really conscious, or whether they just chose it because they liked a certain structure best. (Teacher 4, Meeting 6)

Balancing declarative, procedural and conditional knowledge about text structure proved difficult, and especially conditional knowledge was not yet given sufficient attention. However, teachers did indicate that they saw the importance of balancing these types of knowledge. In Survey 2, two teachers cited it as one of the most important eye-openers, although neither of them indicated that they now applied this in their lessons themselves. During Meeting 6, one of the teachers commented that he felt that goal-oriented attention to declarative, procedural and conditional knowledge should be incorporated in the teaching materials so that teachers do not have to figure it out on their own. Hence, DP4a certainly seems to be viable, but within our project we have only partially succeeded in integrating it properly.

### DP4b. Use of model texts with a clear structure

Since the lesson series introduced the students to various text structures for the first time, we needed texts that could serve as clear examples of a single structure. Similar design studies have indicated that selecting and editing texts can be very time-consuming and thus put a lot of pressure on teachers (Bogaerds-Hazenberg et al., 2019). Therefore, we agreed with the teachers that they could indicate what the content of the texts should be, and that the researchers would provide the texts. The texts had to meet quite a few requirements: they had to be clearly structured, had to fit the topic of the lesson series, and also match the hands-on activities. Therefore, the researchers wrote most of the texts themselves. This was time-consuming.

During Test cycle 2, the teachers were quite satisfied with the texts (Table 6). However, they did indicate that some texts were difficult especially if students’ prior knowledge about the topic was limited or if the text included many unfamiliar words.

DP4b turned out viable since texts with easily identifiable structures were helpful to introduce the various text structures. Because each text was shaped to a single top-level structure and markers of text structures such as signaling words were added, the texts were very appropriate for conveying declarative and procedural knowledge about text structure. However, the fact that the texts had to fit precisely into the context of the lesson series proved a complicating factor in the design process since such texts had to be newly constructed.

### Challenges and benefits of conducting DBR

This section successively reports what teachers’ experiences of the project were in terms of their satisfaction with participation in the project, what they learned from the project, and in what way it led to changes in their teaching practices.

Teachers found it interesting to participate in the project and design their own teaching materials, and noted that it had made them think much more deliberately about their lessons. Doing so strongly differed from only trying out teaching materials, and strengthened a feeling of ownership, as they indicated. This was also endorsed by the remark of a teacher who had not designed her own lessons but only tested teaching materials. In retrospect, she regretted this and would have preferred to be a designer herself if the project were to be continued. Teachers reported to have especially enjoyed working on practical tasks such as creating worksheets or preparing hands-on activities.

A point of interest that teachers mentioned was the overall planning. Teachers stated that six to eight weeks between meetings was in fact too long. High work pressure in primary education and daily issues caused the project to be pushed into the background during the weeks between meetings, and thus induced a decline of attention to the project. On top of that, the COVID-19 pandemic was a major game changer within the process. Logically, the teachers had other things on their mind during periods of school closure and online education, which is why the project was put aside for a while.

Thus, compared to other activities aimed at professionalization, designing their own teaching materials definitely added value for teachers in that they feel a stronger commitment and think much more consciously about their own teaching performance. However, this can only succeed if teachers are sufficiently being facilitated for participation in a design team.

Overall, by the end of the project, teachers felt fairly confident about their mastery of the various elements addressed in the lesson series (Table 8). Teachers most often mentioned the added value of combining hands-on activities with reading and writing activities, and providing text structure instruction during reading and writing activities. When asked whether they had felt a lack of knowledge at certain points, teachers mentioned several examples related to science content, such as building an electric circuit or knowledge about climate change but none about language instruction. Although our data collection was not specifically focused on learning outcomes of the teachers, we can state that all participating teachers gained new insights. Table 9 shows the elements of the lesson series and the number of teachers who state to have implemented them in their own teaching practice. All teachers indicated that they now pay attention to text structure in their lessons, and four teacher make use of graphic organizers. During Meeting 6, Teacher 1 explained that text structure can easily be brought to attention during various lessons:

By explicitly paying attention to these text structures, you can refer back to them later that day. For example, do you remember we were talking about that sequential order, or those signaling words: *first*, and *then* and *finally*? And then, of course, you come across those again later. (Teacher 1, Meeting 6)

**Table 9.** Survey 2 – number of teachers who reported to have implemented a certain instructional feature of the lesson series in one’s own teaching practice (*n* = 6).

Instructional feature	Number of teachers
Drawing attention to specific text structures	6
Using graphic organizers	4
Connecting rhetorical relations with text relations	1
Using hands-on activities to concretize rhetorical relations	2
Making use of authentic reading activities	2
Paying attention to conditional knowledge about text structure	0
Integrating science education and literacy instruction	1

The other elements were only implemented by one or two teachers, and none of the teachers indicated to have implemented conditional knowledge about text structure. Remarkably, despite the fact that teachers were very enthusiastic about the integration of science and literacy and call this a major new insight, only one teacher stated to have entered this to the own classroom.

Teachers plan to use the lesson series again in the next school year or to pass them on to their colleagues. One of the schools would like to expand text structure instruction within the school, and work toward a buildup of learning goals across grades. To accomplish this, teachers indicated that they would need more teaching materials, and especially a wide range of high-quality texts.

## Conclusion and discussion

### Viability of the design principles

The design principles that guided our design process appeared to be viable. It turned out that teaching text structure knowledge can be combined with hands-on learning in science very well, which affirms the synergistic relation between both subjects (Cervetti et al., 2006; Huntley, 1998; Pratt & Pratt, 2004). Comprehension of the concepts taught in science is closely related to the coherence patterns evident in the texts. Obviously, further research is needed to find out to what extent this integrated approach promotes text comprehension and science knowledge. Still, some fine-tuning will be necessary to optimize the teaching materials. This section elaborates on several issues we encountered and provides suggestions for future DBR studies and follow-up research.

In accordance with previous research on text structure instruction (Jones et al., 2016; Meyer & Ray, 2011), using model texts that cover a single top-level structure and match the reading level of the students was found to be an essential component of explicit instruction. Since the texts in our lesson series had to meet quite a few requirements, researchers had to write them, which was time-consuming. To solve this problem one could start by selecting appropriate authentic texts and only then devise hands-on activities and reading-and-writing activities to go with them.

Providing text structure instruction while balancing declarative, procedural and conditional knowledge was completely new to the teachers, which is why they needed additional support. This outcome is consistent with findings of Bogaerds-Hazenberg et al. (2019) in a similar DBR study with teachers in primary education, and calls for teacher professionalization in this area.

We have not yet sufficiently succeeded in embedding the application of conditional knowledge regarding text structure in the teaching materials. Still, it is essential to do this, since students need to be able to flexibly use their metacognitive knowledge about reading strategies in a variety of reading tasks (Lorch et al., 1993; Medina et al., 2021; Paris et al., 1983). Balancing explicit instruction and authentic language activities could serve as a lever in teaching conditional knowledge. In a longer continuum of lessons, emphasis can gradually shift from explicit instruction to more authentic language tasks where students increasingly gain autonomy, and stronger demands are made on conditional knowledge (Iwai, 2011). Further research is needed to find out how this exactly works in text structure instruction.

Although teachers felt best equiped to supervise hands-on activities, it took them a lot of time to prepare and carry out these activities. Moreover, teachers indicated that they felt uncertain about their knowledge regarding several science topics. Therefore, it is recommended to prepare easy-accessible hands-on activities, to provide teachers with all the necessary materials, and make background information available about the science topic of the lessons.

In conclusion, supporting teachers to apply the design principles themselves, and making teaching materials applicable to a wider group of teachers requires professionalization and support. One way to do this could be to design teaching materials as *educative curricular materials*, materials aimed at both student *and* teacher learning, for example by clarifying instructional principles and underlying design principles (Bogaerds-Hazenberg, 2023). Research on the usability of such materials in the field of science education reveals that this seems a promising way to promote teacher professionalization and to ensure that newly acquired knowledge is directly applied in practice (Edelson et al., 2021; Haas et al., 2021).

### DBR in the field of integrated science and literacy education

In response to RQ2, we can conclude that DBR seems quite suitable for bridging the research-practice gap within the field of integrated science-and-literacy education. Although findings from scientific research can be highly relevant to teaching practices, they do not yet render what exactly to teach at a level that is useful for teachers (Seidenberg et al., 2020; Vanderlinde & van Braak, 2010). Actively engaging with the DPs generates high engagement and a sense of ownership (Binkhorst et al., 2017), which was also evident in our study. We noticed that the iteration of multiple design cycles, and applying triangulation in data collection ensured the design to be suitable for educational practice (McKenney & Reeves, 2018).

However, designing educational materials in the field of science-and-literacy integrated education is not an everyday activity for teachers, and in DBR they are invited to apply design principles that they have little knowledge of, and are not experienced with. Therefore, an essential condition for the success of a DBR project that is evident from our results and aligns with related research, is that design teams should receive adequate support in carrying out their design task. This includes organizational support, but also process support and especially support from experts in applying the design principles (Smit et al., 2018; Voogt et al., 2016). A point of consideration here could be the division of labor in the project. Within the range of having teachers only test teaching materials on the one side, or having them design teaching materials from scratch on the other side, various allocations of tasks are possible. For example, teachers could be involved in the design process by generating ideas and providing feedback during the design process, while researchers take care of the design task. This could also make participation in a DBR less demanding for teachers while they are still closely involved in the design process. Despite positive research outcomes toward both language proficiency and science knowledge, the integration of science and literacy is rarely applied in Dutch primary education. Our study shows that DBR can be a useful approach to introduce teachers to this kind of innovation. As in other DBR studies (Binkhorst et al., 2017; Fazio & Gallagher, 2018, 2019), we noticed great engagement and, most importantly, a sense of ownership among participating teachers. Participating in the project changed teachers’ perspective on the integration of science-and-language education, provided them with new insights. However, to continue and further promote the actual use of integrated programs within schools, long-term partnerships between researchers and teachers are needed that allow them to jointly develop and test teaching materials that integrate literacy in the field of science education.
